# Supplementary material for: Single-cell analyses identify monocyte gene expression profiles that influence HIV-1 reservoir size in acutely treated cohorts
Source: Nat Commun. 2025 May 29;16:4975. doi: 10.1038/s41467-025-59833-9 (PMC12122806; doi:10.1038/s41467-025-59833-9)
Supplement: Supplementary file 1 — Supplementary Information [file 41467_2025_59833_MOESM1_ESM.pdf]

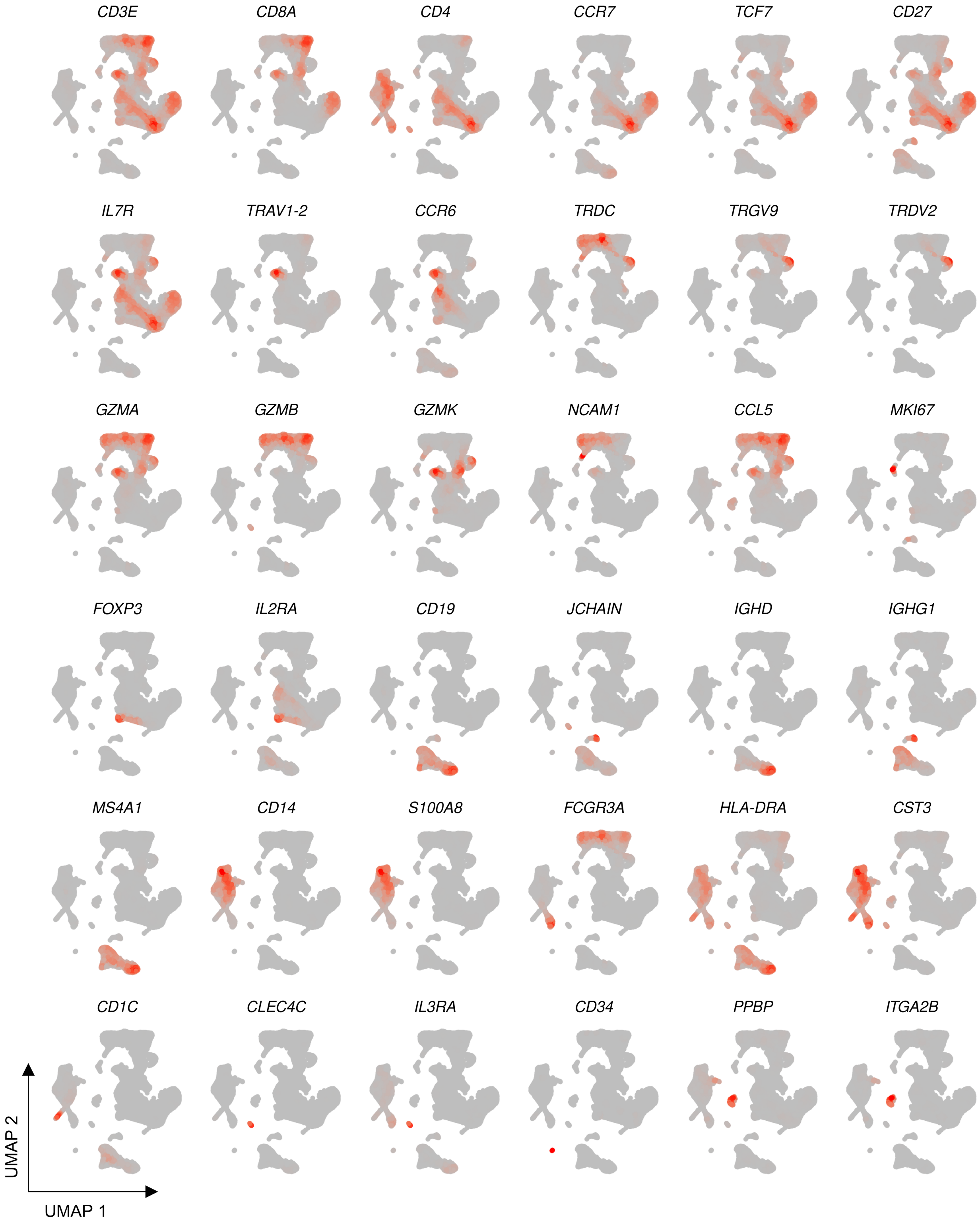

Supplementary Figure 1. Expression of select lineage markers in specific cell subsets. Expression of known lineage marker genes is localized to certain regions on the scRNA-seq UMAP and enables cell type assignment of clusters. Color indicates density of cells expressing the marker and is relative for each gene (gray: no expression and red: highest density of expression).

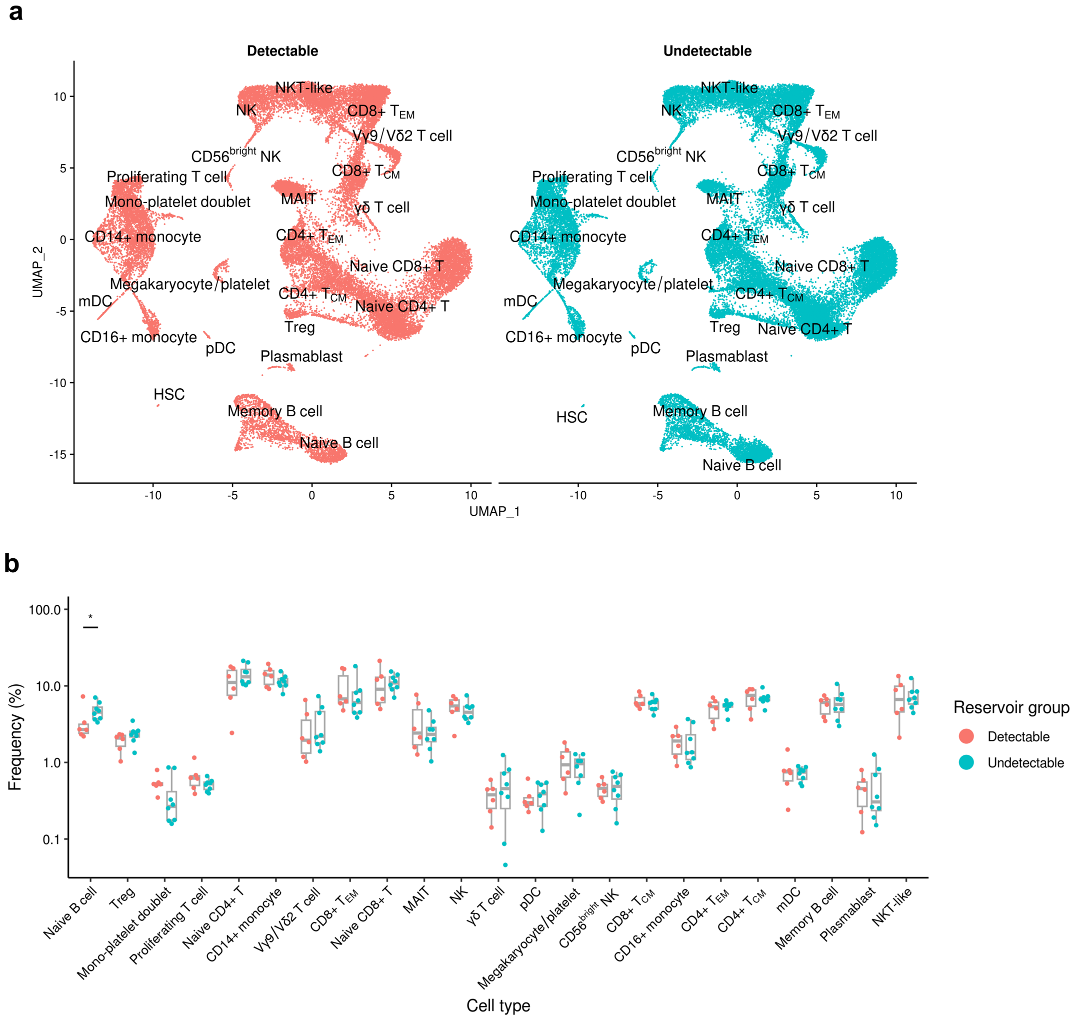

Supplementary Figure 2. Clustering and frequency comparisons of scRNA-seq immune cell subsets. scRNA-seq shows similar **a** spatial patterns of cell populations and **b** frequencies of cells between detectable (red) and undetectable (teal) reservoir groups. Significance was determined by the Mann-Whitney *U* two-sided test, n=14 participants. * nominal $P < 0.05$

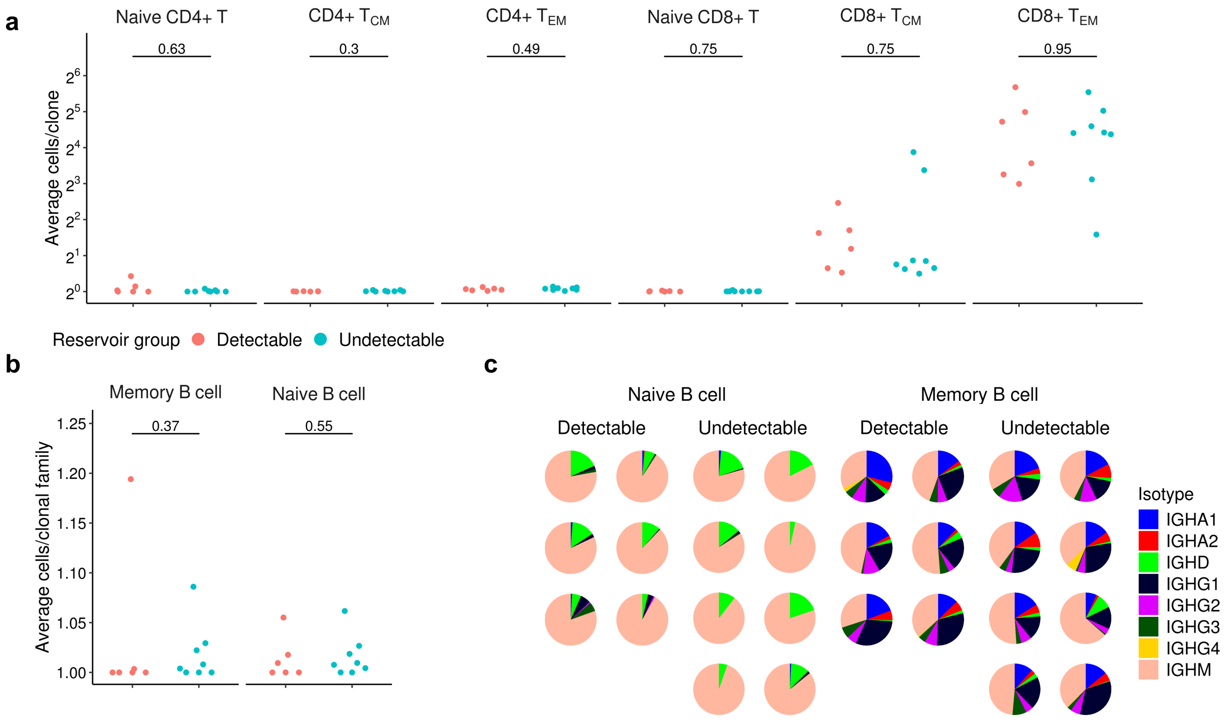

Supplementary Figure 3. T cell receptor and B cell receptor clonal diversity and isotype distribution. Clonal diversity of **a** indicated T cell populations and **b** memory and naive B cell populations. Significance was determined by the Mann-Whitney *U* two-sided test, n=14 participants. **c** BCR isotype distribution of each participant within naïve and memory B cells.

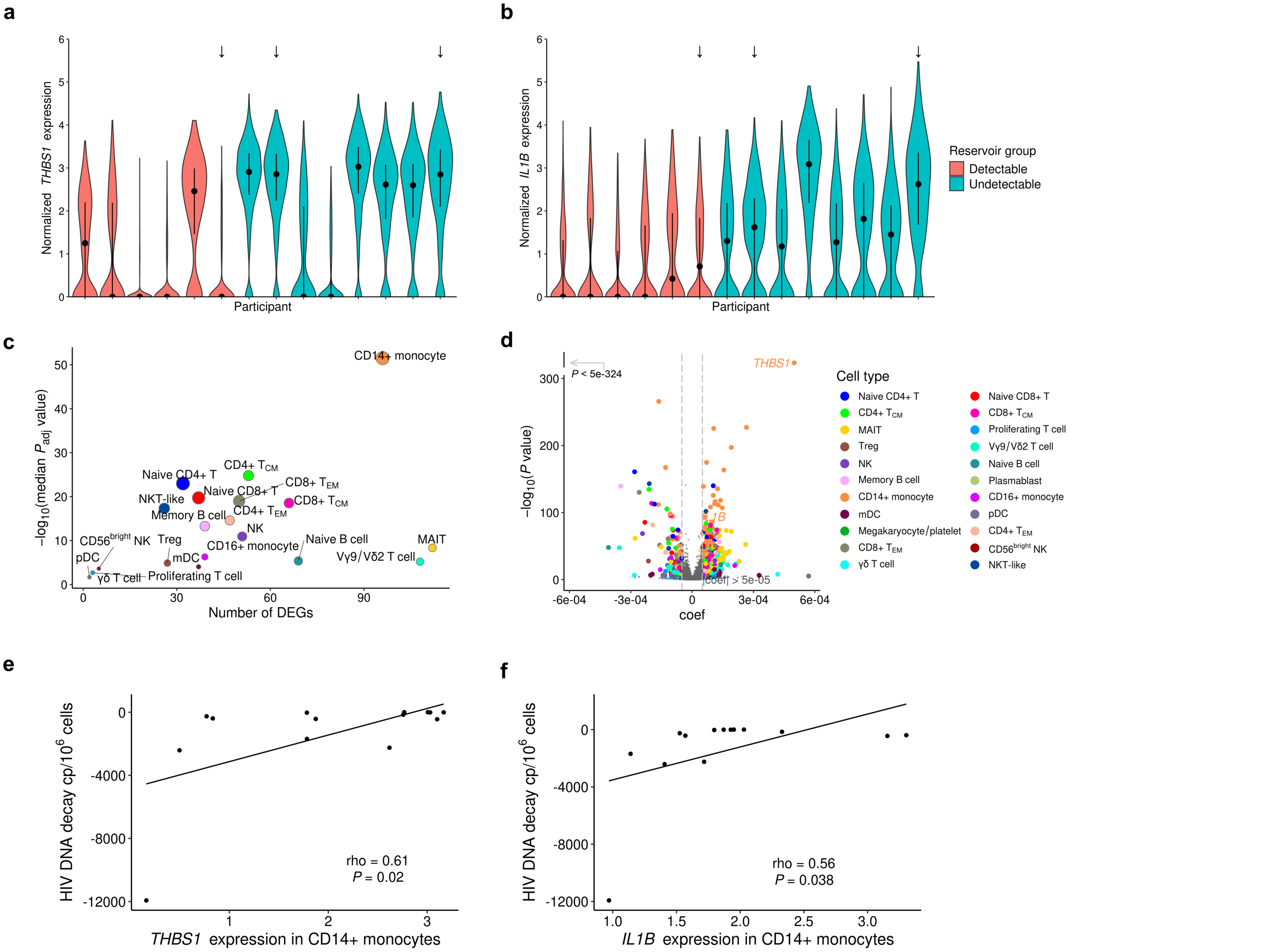

Supplementary Figure 4. Gene expression in CD14+ monocytes across participants in RV254 and HIV DNA outcomes. Gene expression in CD14+ monocytes of **a** *THBS1* and **b** *IL1B* genes across participants based on categorization of total HIV DNA measured from total PBMC or CD4+ T cells. Black circles represent the median values, and vertical lines indicate the interquartile range. Teal: undetectable reservoir; red: detectable reservoir. Three datapoints were missing due to technical differences and insufficient sample to perform the HIV DNA assay in sorted CD4+ T cells and these participants are indicated by vertical arrows. **c, d** Total HIV DNA decay associations when examining change in reservoir from week 0 (AHI) to 48 weeks after ART initiation with **c** number of significant DEG and **d** top genes associating with reservoir decay. Significance was determined using the MAST framework and *P* values shown are adjusted for Bonferroni correction per cell type for the number of expressed genes. DEG summarized in **c** were filtered as described in the Methods section. **e, f** Spearman correlation of total HIV DNA decay with **e** *THBS1* and **f** *IL1B* gene expression in CD14+ monocytes, n=14.

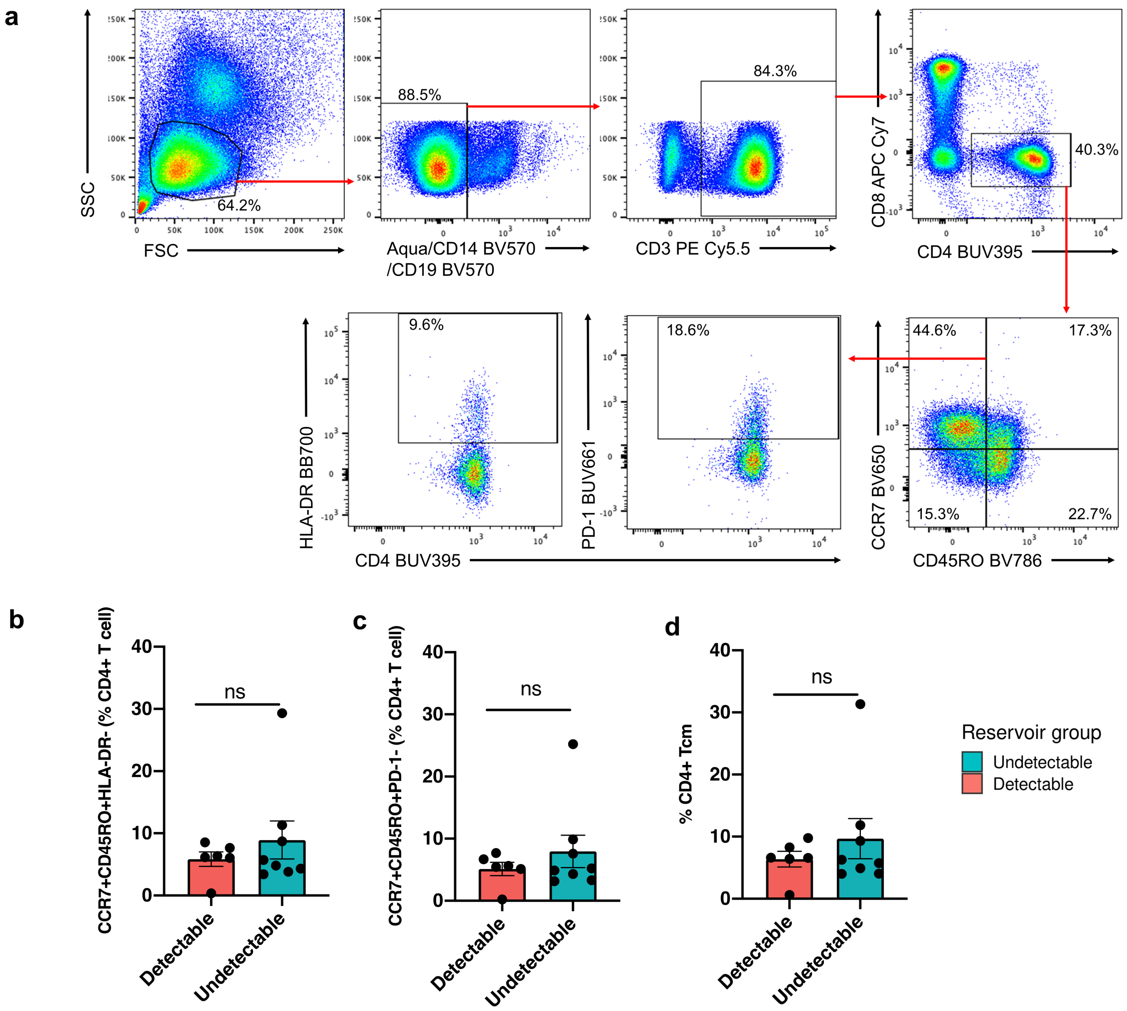

Supplementary Figure 5. No differences in frequencies of CD4+ T_{CM} cell subsets between reservoir groups. a Gating strategy for the identification of CD4+ T_{CM} cell subsets from the RV254 participants. **b** DR- CD4+ T_{CM}, **c** PD-1- CD4+ T_{CM} and **d** CD4+ T_{CM}. Significance was determined by the Mann-Whitney *U* two-sided test, n=14. Teal: undetectable reservoir; red: detectable reservoir. Bar height and error bars represent mean values +/- SEM. Source data are provided in the Source Data file.

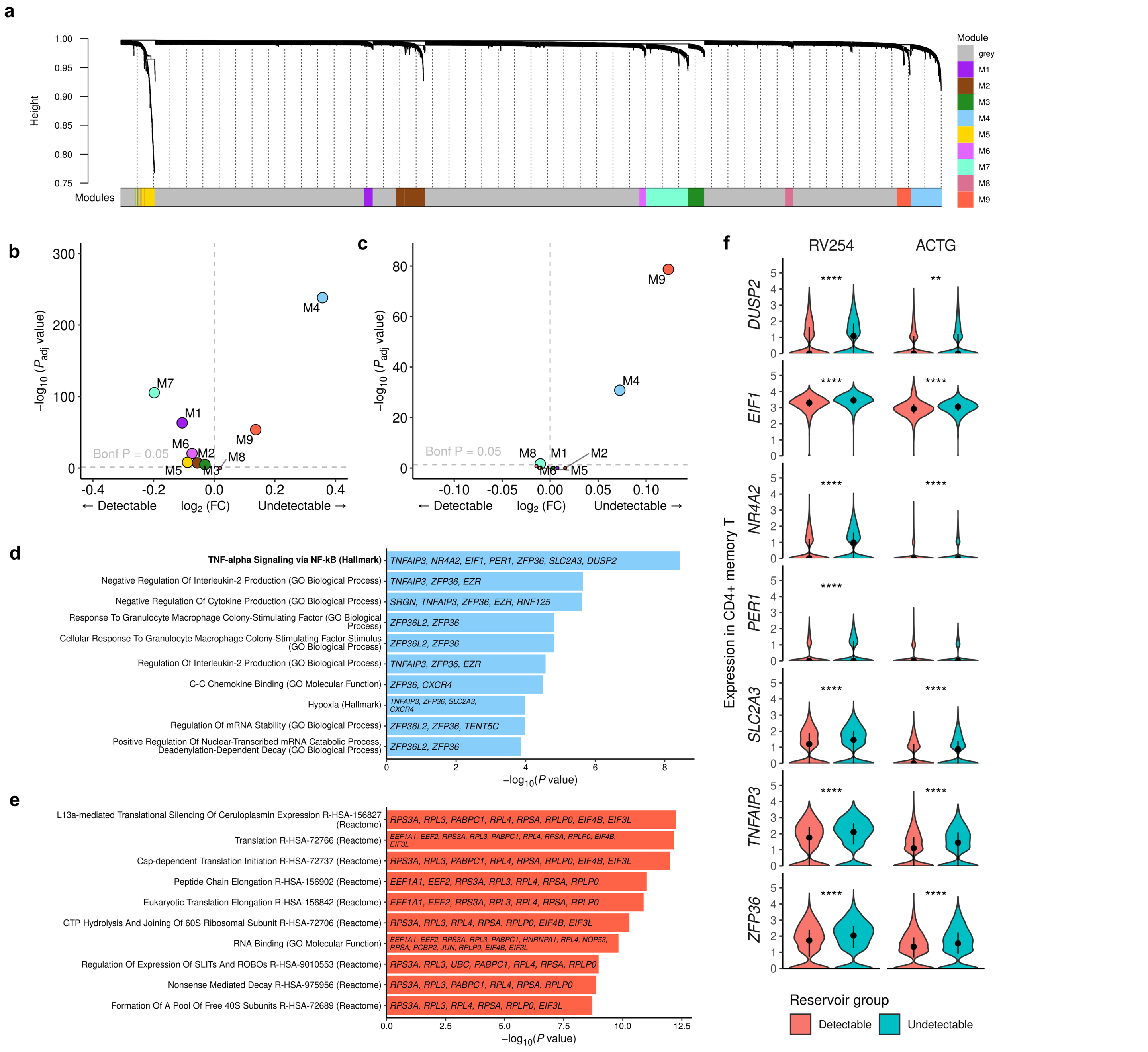

Supplementary Figure 6. Pathway analyses identified distinct signatures associated with reservoir size. a WGCNA dendrogram of modules of coexpressed genes in memory CD4+ T cells from the RV254 Thai study. **b** The M4 and M9 WGCNA modules were enriched in cells from participants with undetectable reservoir (n=8) compared to detectable reservoir (n=6) based on the top 25 hub genes in the module. **c** Module hub genes found in RV254 were enriched in cells from the undetectable reservoir participants in the A5354 cohort when HIV DNA levels were grouped categorically (detectable=12, undetectable=11). Enrichment analysis of **d** M4 and **e** M9 hub genes and pathways significantly enriched (FDR < 0.05). Significance was determined using the EnrichR implementation of Fisher's exact test. *P* values were FDR corrected and the ten most significant genesets per module with adjusted *P* < 0.05 are shown. **f** Violin plots showing significant DEGs in CD4+ memory T cells from the NF-kB pathway genes enriched in the M4 module from both studies. RV254, n=7,536 cells and ACTG, n= 8,331 cells. ** *P* < 0.01, **** *P* < 0.0001. For **b**,**c**, and **f,** significance was determined by the Mann-Whitney *U* two-sided test with Bonferroni correction.

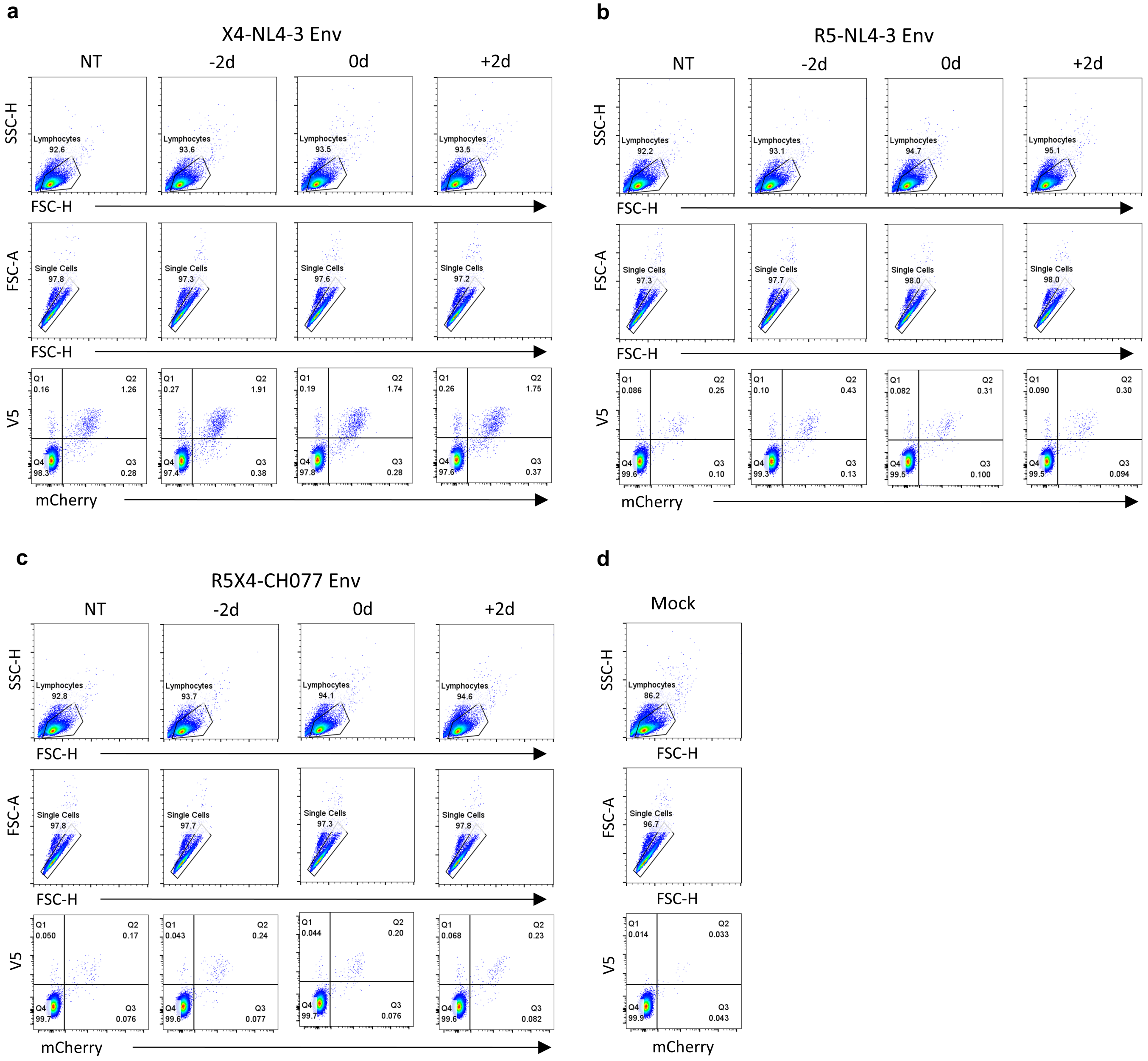

Supplementary Figure 7. Gating strategy for the identification of latent and productive infections in vitro. Latently (V5) and productively (mCherry, V5) infected PBMC with pMorpheus-V5 were pseudo-typed with **a** X4-NL4-3, **b** R5-NL4-3 or **c** R5X4-CH077 viral Env protein. PBMC isolated from healthy participants were cultured with PHA and IL-2 for 3 days, when they were infected with designated viruses. Cultures were treated with IL1B prior, simultaneously or after transduction. Cells were analyzed 3 days post transduction. **d** Mock transduced cells are shown for comparison. NT: no treatment control.

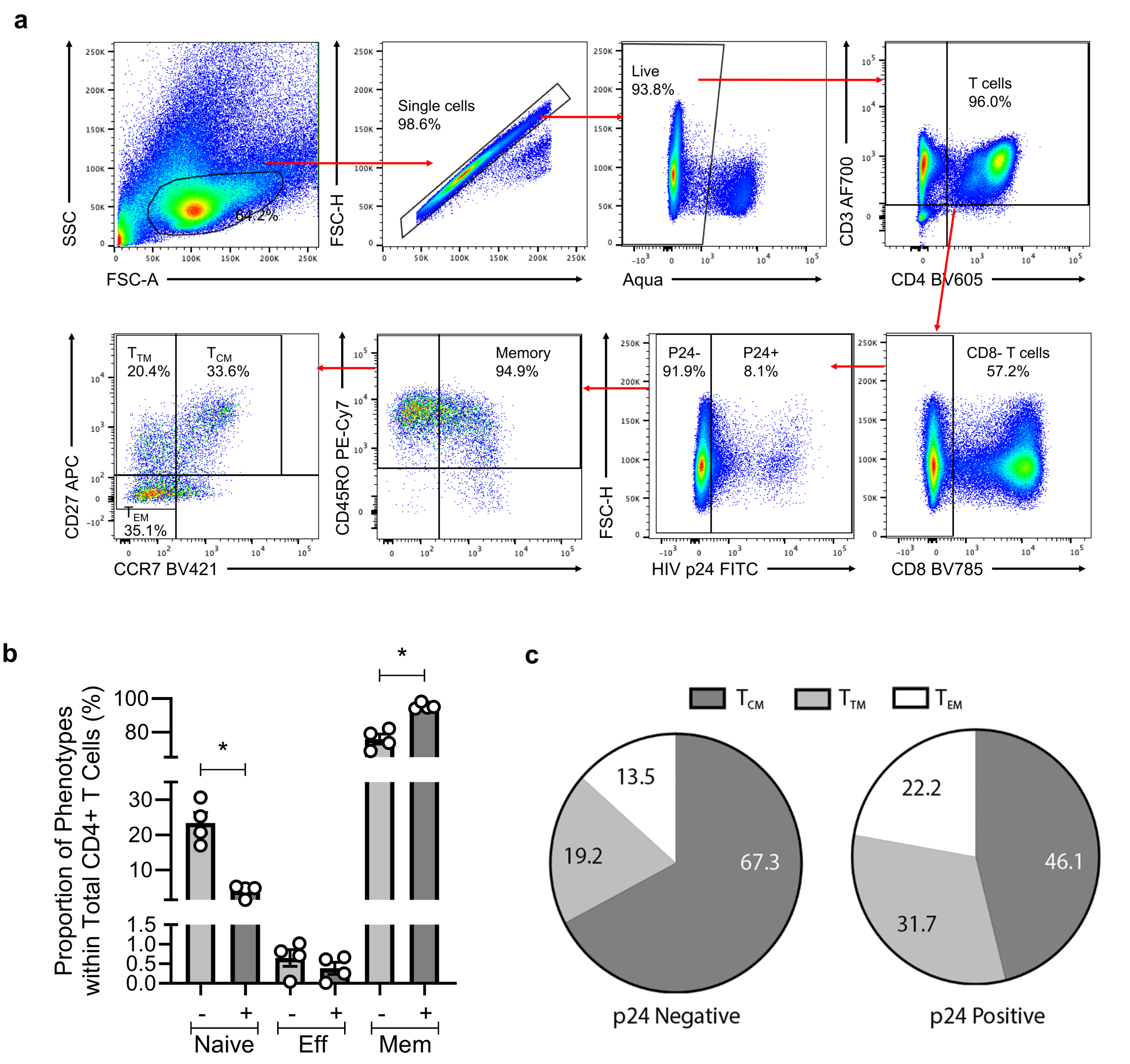

Supplementary Figure 8. Effects of HIV infection on in vitro CD4+ T cell memory phenotypes. a Gating strategy. **b** Bar plot depicts the mean percentages of CD45RO- CCR7- Naive (Naive), CD45RO- CCR7+ Effector (Eff), and CD45RO+ memory (Mem) CD4+ T cell populations in total CD4+ T cells infected or not with HIV, as determined by intracellular p24 staining. Significance was determined using the Mann-Whitney *U* two-sided test. n=4, * $P < 0.05$. Bar height and error bars represent mean values +/- SEM. **c** Pie chart illustrates the mean percentages of memory subsets T_{CM}, T_{TM}, and T_{EM} within total CD45RO+ memory CD4+ T cells. n=4 samples. Source data and exact *P* values are provided in the Source Data file.

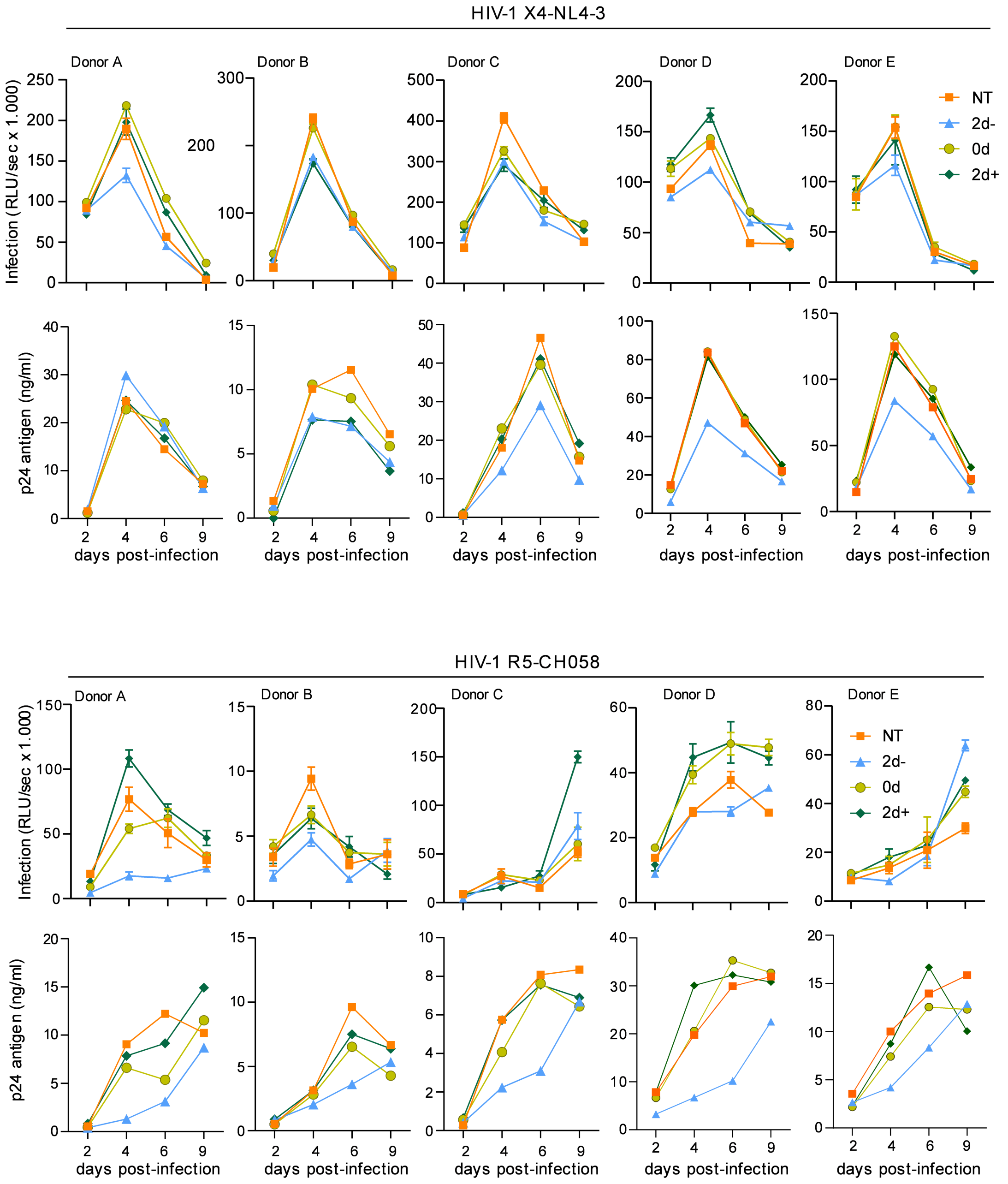

Supplementary Figure 9. Effects of recombinant IL1B on HIV replication in PBMC cultures. Time course plots of the infectious virus and p24 antigen yields of NL4-3 or CH058 (n=5). PBMC were isolated from buffy coats and incubated for 3 days, with PHA and IL-2, when they were infected with NL4-3 or CH058. IL1B (10 ng/ml) was added 2 days prior to infection (2d-), at the same time (0d), or 2 days post-infection (2d+). Supernatants were collected at designated time points and assessed for infectious virus and p24 antigen. Source data are provided in the Source Data file.

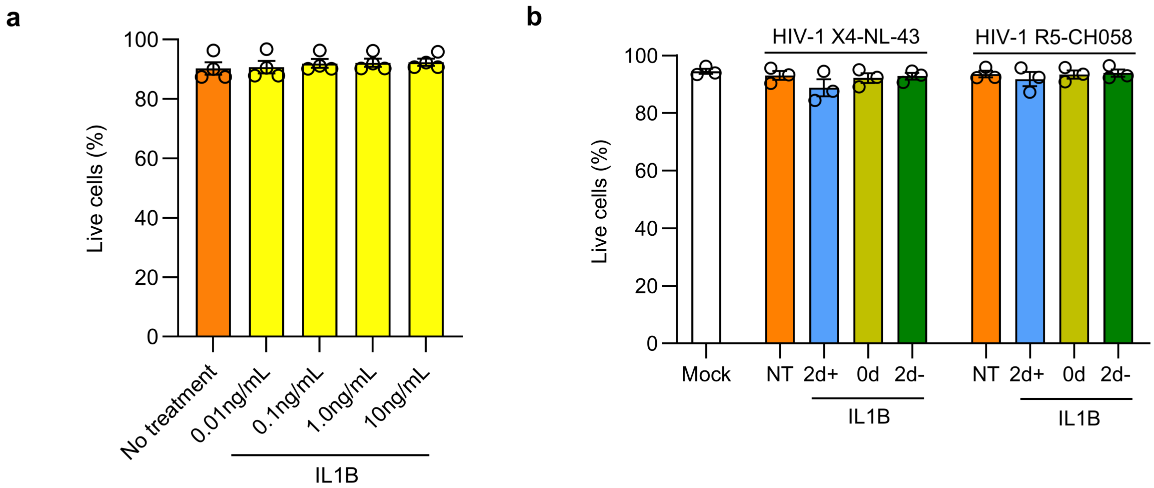

Supplementary Figure 10. Effect of IL1B on cell viability. Isolated PBMC were treated and infected as in **a** Figure 5d (orange: no treatment and yellow: treatment with IL1B; n=4) and **b** 5e-f and Supplementary Figure 9 (white: Mock, orange: no treatment, blue: treatment two days post-infection, yellow: treatment at the same time as infection, green: treatment 2 days prior to infection; n=3), respectively. On day 3 post-infection cells were harvested, stained with fixable viability dye and FACs analyzed for cell viability. Each dot represents a single participant. Bar height and error bars represent mean values +/- SEM. Source data are provided in the Source Data file.

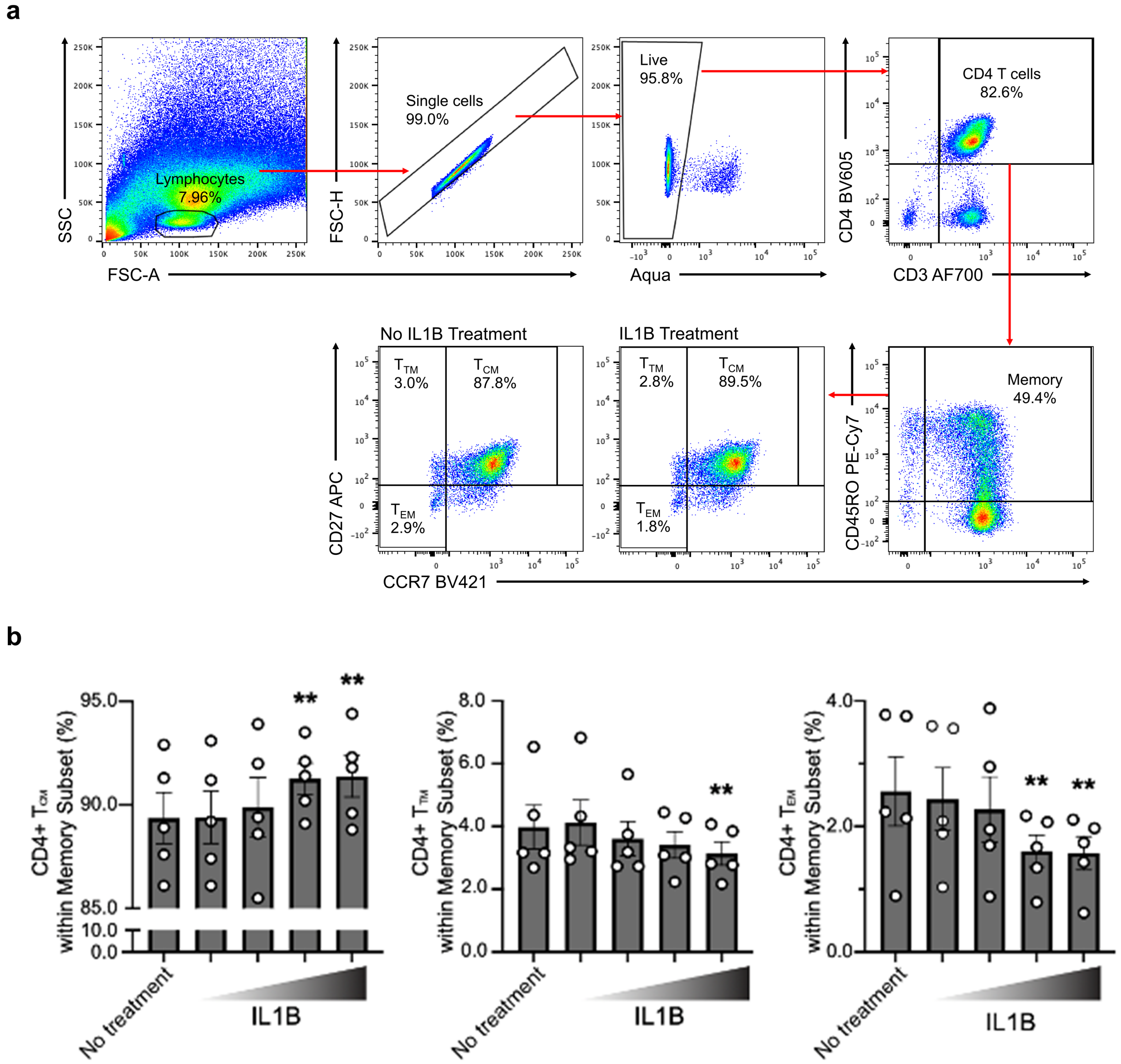

Supplementary Figure 11. Effects of recombinant IL1B on in vitro CD4+ T cell memory phenotypes. a Gating strategy. **b** Percentages of T_{CM}, T_{TM}, and T_{EM} subsets within total memory CD4+ T cells were determined following dose-dependent IL1B treatment at different concentrations (0.01 ng/ml, 0.1 ng/ml, 1 ng/ml, 10 ng/ml). Statistical significance was assessed using the Mann-Whitney *U* two-sided test, based on treated samples relative to their respective untreated control. n=5. ** $P < 0.01$. Bar height and error bars represent mean values +/- SEM. Source data and exact *P* values are provided in the Source Data file.

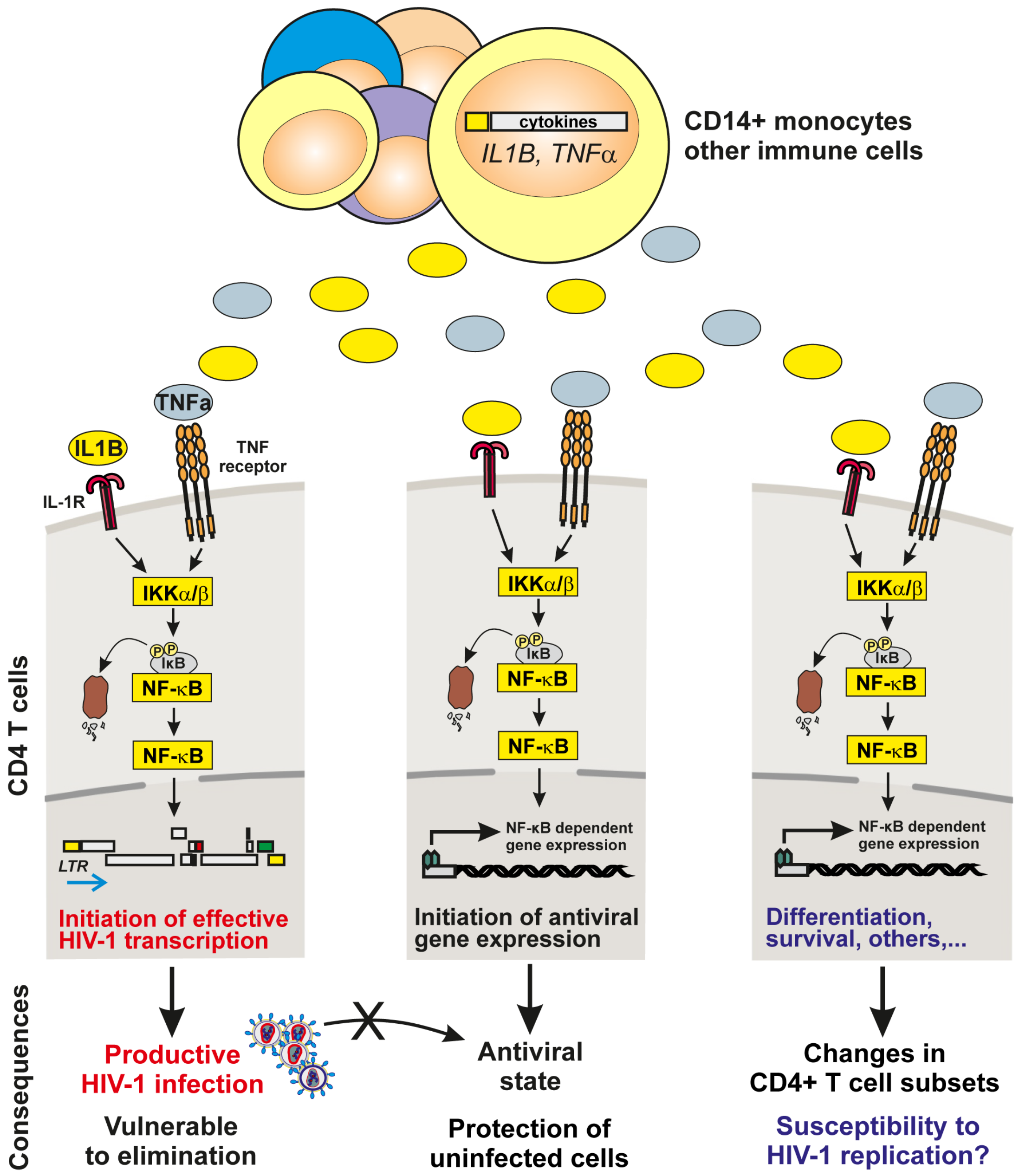

Supplementary Figure 12. Working model of potential mechanisms for IL1B effects on reservoir size. We hypothesize that IL1B may affect the latent HIV reservoir by 1) acting as a natural LRA, 2) contributing to reduced seeding of the reservoir, and 3) changing the composition of CD4+ T cell subsets.

Supplementary table 1. Different cell populations as detected in scRNA-seq analyses.

	RV254		ACTG A5354	
Cell type	Cell count	Frequency	Cell count	Frequency
Naive CD4+ T*	8029	0.128	22289	0.159
CD14+ monocyte**	7853	0.125	17797	0.127
Naive CD8+ T	6860	0.109	12365	0.088
CD8+ T_{EM}	5553	0.088	16740	0.119
NKT-like	4753	0.076	11597	0.083
CD4+ T_{CM}	4315	0.069	-	-
Memory CD4+ T	-	-	13274	0.095
Memory CD4+ T (+IFN)	-	-	361	0.003
CD8+ T_{CM}	3812	0.061	9546	0.068
Memory B cell	3583	0.057	7923	0.057
CD4+ T_{EM}	3221	0.051	-	-
NK	3207	0.051	6241	0.045
Naive B cell	2526	0.040	7301	0.052
MAIT	1975	0.031	2223	0.016
Vγ9/Vδ2 T cell	1868	0.030	555	0.004
Treg	1347	0.021	2652	0.019
CD16+ monocyte	1172	0.019	4425	0.032
Megakaryocyte/platelet	578	0.009	781	0.006
mDC	471	0.007	1524	0.011
Proliferating T cell	358	0.006	607	0.004
Plasmablast	297	0.005	492	0.004
$CD56^{bright}$ NK	294	0.005	934	0.007
γδ T cell	288	0.005	-	-
Mono-platelet doublet	286	0.005	-	-
pDC	228	0.004	352	0.003
HSC	51	0.001	193	0.001

* Naive CD4+ T population in RV254 contained cells from two clusters: Naive CD4+ T, Naive CD4+ T +ifn

** CD14+ monocyte population in RV254 contained cells from two clusters: CD14+ monocyte, CD14+ activated monocyte

Supplementary table 2. Differentially expressed genes in each cell subset (log fold change >|0.25|, Bonferroni $P < 0.05$).

Cell type	Number of DEG*	Genes (Detectable reservoir)	Genes (Undetectable reservoir)
CD14+ monocyte	78	*AC020656.1, BTG2, CCL3L1, CD52, CYBA, EGR2, EGR3, GIMAP7, HLA-DQB1, IER2, JUN, KLF2, MNDA, RGS2, RHOB, TUBA1A, TXNIP, ZFP36L2*	*AC245128.3, ACSL1, ANPEP, AREG, ARL4C, BCL2A1, BCL3, C15orf48, C5AR1, CCL4, CDKN1A, CXCL2, CXCL8, DUSP2, EREG, ETS2, FCAR, FTH1, G0S2, GPR183, GRASP, HIF1A, ICAM1, IL1B, MAFB, MAP3K8, MARCKSL1, NAMPT, NFE2L2, NFKBIA, NFKBIZ, NINJ1, NLRP3, PDE4B, PFKFB3, PHLDA1, PIM3, PLAUR, PLEK, PPIF, PPP1R15A, PRDM1, RAB20, REL, RETN, RNF144B, SAMSN1, SERPINB2, SLC25A37, SOD2, SRGN, THBD, THBS1, TIMP1, TIPARP, TMEM123, TMEM176A, TMEM176B, TNFAIP3, ZNF331*
CD8+ T_{CM}	51	*CCL4, CCL4L2, CD2, CD8B, CMC1, CTSW, CYBA, CYTOR, DYNLL1, GIMAP4, GIMAP7, GZMA, GZMH, HLA-DPB1, HLA-DQA1, HLA-DQB1, LGALS1, NKG7, PLEK, PSMB9, TIGIT*	*ARID5A, CREM, CSRNP1, EZR, FAM177A1, FOSL2, FTH1, GABARAPL1, GADD45B, MTFP1, NFKBIA, NR4A2, PDE4B, PDE4D, PER1, PIK3R1, PTP4A1, SATB1, SELENOK, SLC7A5, SRGN, SYTL3, TNFAIP3, TSPYL2, TUBA4A, USP36, YPEL5, ZFP36, ZFP36L2, ZNF331*
CD8+ T_{EM}	46	*ANXA1, CCL4L2, CD3D, CD3G, CD8B, CLIC3, CX3CR1, CYTOR, DYNLL1, EMP3, GIMAP4, GIMAP7, GZMB, LAIR2, LINC00861, M6PR, MT2A, PLEK, PRF1, PSMB9, STAT1, STK38*	*CREM, CXCR4, DNAJB1, DUSP2, FOSL2, GADD45B, GNLY, IL7R, KLRB1, KLRC3, MAP3K8, NR4A2, PER1, PIK3R1, RBM38, SLC7A5, SYTL3, TNFAIP3, TUBA4A, TYROBP, ZFP36, ZFP36L2, ZNF331, ZNF683*
CD16+ monocyte	38	*CD52, CLEC12A, CX3CR1, DUSP6, DYNLL1, GIMAP7, HLA-DQA2, SMIM25, TNFSF10, TXNIP*	*ADK, CCL3, CCL4, CD83, CXCL8, DUSP2, ETS2, G0S2, ICAM1, IL1B, NFKBIA, NINJ1, NLRP3, PDE4B, PFKFB3, PIM3, PLAUR, PLEK, PPP1R15A, REL, RETN, SAMSN1, SNHG15, TMEM176A, TMEM176B, TNF, TNFAIP3, ZFP36L1*
Vγ9/Vδ2 T cell	36	*CCL3, CCL4L2, CX3CR1, CYTOR, DENND2D, GIMAP7, IL7R, KLF6, MYOM2, PLEK, TRAC, TXNIP*	*ARID5A, CREM, CSRNP1, CXCR4, DUSP2, FOSL2, FTH1, GABARAPL1, GADD45B, IFNGR1, LDHA, MAP3K8, METRNL, NFE2L2, NR4A2, PDE4B, PER1, PIK3R1, SELENOK, SLC7A5, SRSF7, TNFAIP3, TUBA4A, ZNF331*
mDC	33	*DYNLL1, S100A11, TXNIP*	*ATP1B3, CDKN1A, CREM, CSRNP1, CXCL16, DUSP2, DUSP4, FTH1, GPR183, HERPUD1, INSIG1, JARID2, MAP3K8, NAMPT, NFE2L2, NR4A2, NR4A3, PFKFB3, PLAUR, RASSF5, REL, RILPL2, SAMSN1, SLC2A3, SNX9, SRGN, STX11, THBS1, TIPARP, ZNF331*
NK	32	*CX3CR1, DENND2D, DYNLL1, GIMAP7, LAIR2, LINC00861, PSMB9, S100A11, UCP2, ZNF600*	*AREG, CCL4, CCL4L2, CD3E, COL6A2, CREM, CSNK1D, CSRNP1, CXCR4, DIP2A, DUSP2, GABARAPL1, GADD45B, IFNGR1, MAP3K8, NFKBIA, NR4A2, PER1, PIK3R1, S100B, ZFP36L2, ZNF331*
MAIT	28	*CCL4, CTSW, GBP5, GIMAP4, GIMAP7, KLRF1, STAT1*	*BHLHE40, CREM, CXCR4, DNAJB1, FTH1, GTF3C1, HIST1H1C, LITAF, MAP3K8, NR4A2, PDE4B, PDE4D, PER1, PIK3R1, RNF125, SLC7A5, SRGN, SRSF7, SYTL3, TNFAIP3, ZNF331*
NKT-like	25	*CD3D, CD3G, CD8A, CD8B, CX3CR1, CYTOR, DYNLL1, IKZF2, LAIR2, TRAC, TRGC2*	*ALOX5AP, CMC1, COL6A2, FGFBP2, HLA-DRB1, KIR3DL1, KLRB1, MGAT4A, NFKBIA, PRMT2, PTGDS, SYNGR1, TXK, TYROBP*
CD4+ T_{CM}	23	*GIMAP4, GIMAP7, S100A11*	*ARID5A, CHD1, CREM, DNAJB1, EZR, FTH1, H3F3B, NR3C1, NR4A2, PER1, PIK3IP1, PIK3R1, SARAF, SESN3, SRGN, TENT5C, YPEL5, ZFP36, ZFP36L2, ZNF331*
Memory B cell	17	*HLA-DQA2*	*ARID5A, CD55, CD83, CXCR4, EZR, GRASP, HERPUD1, HIF1A, LY9, MAPK1IP1L, NFKBIA, NFKBID, NR4A1, PELI1, SNX9, ZNF331*
Naive B cell	14	*CD79B, CPNE5, CYBA, FCRL3, HLA-DQA2, PSMB9, SCIMP*	*EZR, HLA-DQA1, NFKBIA, PELI1, PIK3IP1, SESN1, SNX9*
CD4+ T_{EM}	14	*GIMAP7, S100A11*	*ARID5A, CREM, FTH1, LITAF, NR4A2, PDE4D, PER1, PIK3R1, RNF125, SRGN, ZFP36L2, ZNF331*

Naive CD4+ T	9	*CYBA*	*ARID5A, DNAJB1, PIK3IP1, SARAF, SOCS3, TOB1, ZFP36L2, ZNF331*
Treg	7	*DYNLL1, S100A11*	*PELI1, PIK3IP1, PIK3R1, SARAF, SBDS*
Naive CD8+ T	6	*CYBA, LINC01871*	*ARID5A, DCXR, FTH1, ZNF331*
pDC	3	*CYBA, HLA-A, HLA-DQB1*	
CD56^{bright} NK	3		*FTL, H3F3B, LITAF*
Plasmablast	1	*GSTM1*	
γδ T cell	1		*FTH1*
Megakaryocyte/platelet	0		
Proliferating T cell	0		

* Top two significant DEG are highlighted in red, *not including genes that are blacklisted or expressed in less than 10% of cells in both groups

Supplementary table 3. Top 20 enriched pathways and processes.

Term	Description	Log(p-value)	Log(q-value)	InTerm_InList	Genes*
GO:0046649	lymphocyte activation	-20.9	-16.6	42/748	*ANXA1, BCL3, PRDM1, ZFP36L1, ZFP36L2, CD2, CD3D, CD3E, CD3G, CD8A, CD8B, CD79B, CDKN1A, KLF6, MAP3K8, CD55, GPR183, EGR3, HLA-A, HLA-DPB1, ICAM1, IL1B, IL7R, LGALS1, LY9, MNDA, PIK3R1, PRF1, SATB1, TNFAIP3, TYROBP, CD83, MAFB, PELI1, SELENOK, SAMSN1, NFKBIZ, NFKBID, NLRP3, FCRL3, TIGIT, ZNF683*
GO:0001817	regulation of cytokine production	-19.9	-15.9	41/755	*ANXA1, BCL3, C5AR1, CD2, CD3E, CX3CR1, CYBA, EREG, HIF1A, HLA-A, HLA-DPB1, IFNGR1, IL1B, LY9, MNDA, PDE4B, PDE4D, PER1, PIK3R1, SRGN, REL, CCL3, STAT1, THBS1, TNF, TNFAIP3, TXK, TYROBP, EZR, ZFP36, NR4A3, CD83, LITAF, KLF2, RNF125, PELI1, SELENOK, NLRP3, GBP5, TIGIT, SCIMP*
GO:0019221	cytokine-mediated signaling pathway	-14.9	-11.8	36/796	*ANXA1, CDKN1A, MAP3K8, CX3CR1, EREG, ACSL1, CXCL2, HIF1A, HLA-A, HLA-DPB1, HLA-DQA1, HLA-DQA2, HLA-DQB1, HLA-DRB1, ICAM1, IFNGR1, IL1B, IL7R, CXCL8, MT2A, NFKBIA, SERPINB2, PIK3R1, PSMB9, CCL3, CCL3L1, CCL4, SOD2, STAT1, TIMP1, TNF, TNFAIP3, TXK, CXCR4, SOCS3, PELI1*
GO:0002366	leukocyte activation involved in immune response	-10.9	-8.2	29/711	*ANPEP, ANXA1, BCL3, C5AR1, CYBA, CD55, GPR183, FCAR, FTH1, FTL, ICAM1, LGALS1, LY9, MNDA, PLAUR, S100A11, CCL3, SLC2A3, TYROBP, NR4A3, DYNLL1, SYNGR1, YPEL5, RETN, NFKBIZ, NFKBID, NLRP3, CLEC12A, ZNF683*
GO:1902532	negative regulation of intracellular signal transduction	-12.5	-9.6	28/571	*BCL3, DUSP2, DUSP4, DUSP6, HIF1A, IL1B, NFE2L2, PDE4D, PER1, PLAUR, PLEK, RGS2, SOD2, STAT1, THBS1, TNFAIP3, EZR, SOCS3, LITAF, HERPUD1, PPIF, STK38, SESN1, RNF125, NFKBID, PIK3IP1, NLRP3, SESN3*
GO:0043068	positive regulation of programmed cell death	-9.8	-7.3	27/688	*ANXA1, RHOB, CDKN1A, DUSP6, GZMA, GZMB, PRMT2, JUN, LDHA, MNDA, GADD45B, PLAUR, S100B, CCL3, SOD2, THBS1, TNF, TYROBP, NR4A3, TNFSF10, PPIF, TXNIP, PHLDA1, G0S2, NFKBID, NLRP3, SCIMP*
GO:0097190	apoptotic signaling pathway	-11.1	-8.3	27/607	*BCL2A1, BCL3, CD3E, CDKN1A, CX3CR1, GZMH, GZMB, HIF1A, ICAM1, IL1B, JUN, NFE2L2, NR4A2, PIK3R1, PLAUR, SRGN, SOD2, THBS1, TNF, TNFAIP3, TYROBP, TNFSF10, HERPUD1, PPIF, PPP1R15A, G0S2, SELENOK*
GO:0071396	cellular response to lipid	-10.9	-8.2	27/616	*ANXA1, ZFP36L1, ZFP36L2, CX3CR1, CD55, NR3C1, CXCL2, NR4A1, PRMT2, ICAM1, IL1B, CXCL8, INSIG1, NFKBIA, NR4A2, PDE4B, PDE4D, PER1, CCL3, TNF, TNFAIP3, ZFP36, NR4A3, LITAF, TRAC, NLRP3, SCIMP*
GO:1901652	response to peptide	-10.0	-7.4	24/534	*ANXA1, AREG, ZFP36L1, CYBA, EGR2, EREG, NR4A1, ICAM1, IL1B, NFE2L2, NFKBIA, NR4A2, PIK3R1, STAT1, TIMP1, TNFAIP3, BTG2, NR4A3, TNFSF10, SOCS3, NAMPT, KLF2, RETN, SESN3*
GO:0050900	leukocyte migration	-10.5	-7.8	24/504	*ANXA1, ATP1B3, C5AR1, CD2, CX3CR1, GPR183, CXCL2, ICAM1, IL1B, CXCL8, PDE4B, PDE4D, PIK3R1, CCL3, CCL3L1, CCL4, THBD, THBS1, TNF, CXCR4, SLC7A5, SBDS, CXCL16, SELENOK*
GO:0002683	negative regulation of immune system process	-10.9	-8.2	24/483	*ANXA1, ZFP36L1, CD55, IL7R, MNDA, NFE2L2, NFKBIA, PIK3R1, CCL3, THBS1, TNF, TNFAIP3, TYROBP, EZR, ZFP36, MAFB, TMEM176B, RNF125, TMEM176A, PELI1, SAMSN1, NFKBID, FCRL3, TIGIT*
GO:0009636	response to toxic substance	-10.0	-7.4	24/533	*ALOX5AP, ANXA1, AREG, RHOB, CDKN1A, GSTM1, ICAM1, JUN, LDHA, MT2A, NFE2L2, NR4A2, RGS2, CCL3, CCL4, SOD2, STAT1, TNF, TNFAIP3, NR4A3, PPIF, KLF2, TXNIP, SESN1*
GO:0032496	response to lipopolysaccharide	-10.5	-7.8	20/338	*C5AR1, CX3CR1, CD55, CXCL2, ICAM1, IL1B, CXCL8, JUN, NFKBIA, PDE4B, PDE4D, CCL3, THBD, TNF, TNFAIP3, ZFP36, LITAF, PELI1, NLRP3, SCIMP*
hsa04640	Hematopoietic cell lineage	-15.4	-12.1	16/97	*ANPEP, CD2, CD3D, CD3E, CD3G, CD8A, CD8B, CD55, HLA-DPB1, HLA-DQA1, HLA-DQA2, HLA-DQB1, HLA-DRB1, IL1B, IL7R, TNF*
M54	PID IL12 2PATHWAY	-15.4	-12.1	14/63	*CD3D, CD3E, CD3G, CD8A, CD8B, GZMA, GZMB, HLA-A, HLA-DRB1, IL1B, GADD45B, CCL3, CCL4, STAT1*
hsa05142	Chagas disease (American trypanosomiasis)	-9.9	-7.4	12/102	*CD3D, CD3E, CD3G, IFNGR1, IL1B, CXCL8, JUN, NFKBIA, PIK3R1, CCL3, CCL3L1, TNF*
GO:0071216	cellular response to biotic stimulus	-13.2	-8.8	11/249	*CX3CR1, ICAM1, IL1B, CXCL8, NFKBIA, PDE4B, CCL3, TNF, TNFAIP3, TXNIP, NLRP3*
GO:0002757	immune response-activating signal transduction	-12.8	-8.8	10/647	*CD79B, CYBA, HLA-DQA1, HLA-DQA2, NFKBIA, PSMB9, EZR, PELI1, FCRL3, SCIMP*
GO:0033077	T cell differentiation in thymus	-9.0	-6.7	10/72	*ZFP36L1, ZFP36L2, CD3D, CD3E, CD3G, EGR3, IL1B, IL7R, MAFB, NFKBID*
R-HSA-6783783	Interleukin-10 signaling	-10.1	-6.8	6/47	*ICAM1, IL1B, CXCL8, CCL3, CCL4, TNF*

*Genes from the input DEG lists that are present in the given enriched pathway/process are colored as follows: Teal - upregulated in the undetectable group in all cell subsets in which it is a DEG; red - upregulated in the detectable group; black - upregulated in both groups in different cell subsets

Supplementary table 4. Enriched pathways for each reservoir phenotype stratified by cell subset (NES ≥|1.4|, $P < 0.001$).

Cell type	Enrichment in phenotype	Geneset	Size	NES
CD14+ monocyte	undetectable	R-HSA-6783783: Interleukin-10 signaling	43	2.3
CD16+ monocyte	undetectable	R-HSA-6783783: Interleukin-10 signaling	43	2.0
CD14+ monocyte	undetectable	GO:0032496: response to lipopolysaccharide	260	1.9
CD14+ monocyte	undetectable	GO:0071216: cellular response to biotic stimulus	198	1.8
pDC	undetectable	GO:0050900: leukocyte migration	414	1.6
mDC	undetectable	GO:0032496: response to lipopolysaccharide	260	1.5
CD14+ monocyte	undetectable	GO:0050900: leukocyte migration	414	1.5
CD16+ monocyte	undetectable	GO:0002683: negative regulation of immune system process	387	1.5
NK	undetectable	GO:1902532: negative regulation of intracellular signal transduction	433	1.5
CD4+ T_{EM}	undetectable	GO:1902532: negative regulation of intracellular signal transduction	433	1.4
CD4+ T_{CM}	undetectable	GO:0071396: cellular response to lipid	478	1.4
MAIT	undetectable	GO:0002683: negative regulation of immune system process	387	1.4
MAIT	undetectable	GO:1902532: negative regulation of intracellular signal transduction	433	1.4
CD14+ monocyte	undetectable	GO:0019221: cytokine-mediated signaling pathway	668	1.4
CD14+ monocyte	undetectable	GO:0071396: cellular response to lipid	478	1.4
CD16+ monocyte	undetectable	GO:0001817: regulation of cytokine production	618	1.4
CD16+ monocyte	undetectable	GO:0032496: response to lipopolysaccharide	260	1.4
Naive CD4+ T	detectable	GO:0046649: lymphocyte activation	645	-1.4
Naive CD4+ T	detectable	GO:0002757: immune response-activating signal transduction	444	-1.4

Supplementary table 5. Associations of increased *THBS1*/*IL1B* expression with cell subset population frequencies obtained by flow cytometry, and their effects on reservoir size.

Phenotypic frequency	Gene	Beta	Unadjusted *P*	Notes
CD4+ CCR7+ CD45RO+ HLA-DR-	*IL1B*	-82.53	0.003	central memory
CD4+ CCR7+ CD45RO- PD-1+	*IL1B*	-223.9	0.003	exhausted naïve
Ki67+ (% of CD56 dim NK cells)	*THBS1*	-44.19	0.01	
CD163+ classical	*IL1B*	-22.06	0.01	classical monocytes
CD4+ CCR7+ CD45RO+ HLA-DR-	*THBS1*	-30.52	0.01	central memory
CD4+ CCR7+ CD45RO+ PD-1+	*IL1B*	-318.65	0.01	exhausted central memory
CD4+ CCR7+ CD45RO+ PD-1-	*THBS1*	-33.07	0.01	central memory
CD25+ (% of CD4)	*IL1B*	-12.34	0.01	
Resting memory (% of B cells)	*IL1B*	-8.73	0.02	
CD4+ CCR7+ CD45RO+ PD-1-	*IL1B*	-75.24	0.02	central memory
CD39+ CCR5+ (% of CD8)	*IL1B*	-129.5	0.02	
CD39+ (% of CD4)	*IL1B*	-16.37	0.02	
CD69+ (% of CD4)	*IL1B*	-356.67	0.03	
Classical monocytes	*IL1B*	-26.76	0.04	
CD38+ (% of CD56 dim NK cells)	*IL1B*	-4.83	0.04	
CD36+ classical	*IL1B*	-28.95	0.04	classical monocytes
CD4+ CD25+ CD127- HLA-DR+	*IL1B*	-90.96	0.04	activated Tregs
Ki67+ (% of CD4)	*IL1B*	-125.16	0.04	
CD27+ (% of B cells)	*IL1B*	-8.06	0.04	
CD13+ classical	*IL1B*	-26.8	0.05	classical monocytes

Overlapping associations between *THBS1* and *IL1B* are shown in red.
