# Supplementary material for: Single-cell analyses identify monocyte gene expression profiles that influence HIV-1 reservoir size in acutely treated cohorts
Source: Nat Commun. 2025 May 29;16:4975. doi: 10.1038/s41467-025-59833-9 (PMC12122806; doi:10.1038/s41467-025-59833-9)
Supplement: Supplementary file 3 — Description of Additional Supplementary Files [file 41467_2025_59833_MOESM3_ESM.pdf]

Description of Additional Supplementary Files

File Name: Supplementary Data 1
Description: Demographics and clinical data of participants from the study.

File Name: Supplementary Data 2
Description: Cell populations assessed by flow cytometry of surface marker expression.

File Name: Supplementary Data 3
Description: List of antibodies.
