# Supplementary material for: Single-cell analyses identify monocyte gene expression profiles that influence HIV-1 reservoir size in acutely treated cohorts
Source: Nat Commun. 2025 May 29;16:4975. doi: 10.1038/s41467-025-59833-9 (PMC12122806; doi:10.1038/s41467-025-59833-9)
Supplement: Supplementary file 7 — Reporting Summary [file 41467_2025_59833_MOESM7_ESM.pdf]

nature portfolio

Corresponding author(s): Rasmi Thomas

Last updated by author(s): Mar 3, 2025

Reporting Summary

Nature Portfolio wishes to improve the reproducibility of the work that we publish. This form provides structure for consistency and transparency in reporting. For further information on Nature Portfolio policies, see our Editorial Policies and the Editorial Policy Checklist.

Statistics

For all statistical analyses, confirm that the following items are present in the figure legend, table legend, main text, or Methods section.

n/a	Confirmed	
☐	☒	The exact sample size (*n*) for each experimental group/condition, given as a discrete number and unit of measurement
☐	☒	A statement on whether measurements were taken from distinct samples or whether the same sample was measured repeatedly
☐	☒	The statistical test(s) used AND whether they are one- or two-sided<br>*Only common tests should be described solely by name; describe more complex techniques in the Methods section.*
☐	☒	A description of all covariates tested
☐	☒	A description of any assumptions or corrections, such as tests of normality and adjustment for multiple comparisons
☐	☒	A full description of the statistical parameters including central tendency (e.g. means) or other basic estimates (e.g. regression coefficient) AND variation (e.g. standard deviation) or associated estimates of uncertainty (e.g. confidence intervals)
☐	☒	For null hypothesis testing, the test statistic (e.g. *F*, *t*, *r*) with confidence intervals, effect sizes, degrees of freedom and *P* value noted<br>*Give P values as exact values whenever suitable.*
☒	☐	For Bayesian analysis, information on the choice of priors and Markov chain Monte Carlo settings
☒	☐	For hierarchical and complex designs, identification of the appropriate level for tests and full reporting of outcomes
☒	☐	Estimates of effect sizes (e.g. Cohen's *d*, Pearson's *r*), indicating how they were calculated

Our web collection on statistics for biologists contains articles on many of the points above.

Software and code

Policy information about availability of computer code

Data collection	Detailed Information provided in the methods section. PBMC suspensions for single cell sequencing were processed using the 10x Genomics Chromium Controller and sequencing data was collected using a NovaSeq 6000 sequencing instrument (Illumina) using the NovaSeq Control Software, version 1.7.5. Flow cytometry data was collected BD FACSAria II SORP Cell Sorter using BD FACS Diva software (version 8.0.1) or a ThermoFisher BigFoot Spectral Cell Sorter using ThermoFisher SQS software (version 1.13.21278.0)
Data analysis	All descriptive and inferential statistical analyses were performed using R 3.4.1 GUI 1.70 build (7375) v3.0 and higher, and GraphPad Prism 8.0 statistical software packages (GraphPad Software, La Jolla CA). Code is provided for all figures in the paper in the Figshare database.

For manuscripts utilizing custom algorithms or software that are central to the research but not yet described in published literature, software must be made available to editors and reviewers. We strongly encourage code deposition in a community repository (e.g. GitHub). See the Nature Portfolio guidelines for submitting code & software for further information.

Data

Policy information about availability of data

All manuscripts must include a data availability statement. This statement should provide the following information, where applicable:

- Accession codes, unique identifiers, or web links for publicly available datasets
- A description of any restrictions on data availability
- For clinical datasets or third party data, please ensure that the statement adheres to our policy

All gene expression data has been submitted to the GEO repository with accession number (GSE220790, GSE256089) and links to access are provided in the data availability document. Code is available at https://doi.org/10.6084/m9.figshare.c.7074125.

Research involving human participants, their data, or biological material

Policy information about studies with human participants or human data. See also policy information about sex, gender (identity/presentation), and sexual orientation and race, ethnicity and racism.

Reporting on sex and gender	Sex and gender information is described in the methods section and extended table 1 where available. No sex- and gender-based analyses was possible because in both studies >90% of participants were cis-male. The abstract includes mention of the study being performed only in males due to the distribution of the cohorts.
Reporting on race, ethnicity, or other socially relevant groupings	Provided in the methods section and data table 1.
Population characteristics	Provided in the methods section and extended tables.
Recruitment	Not applicable.
Ethics oversight	All participants from the aforementioned human studies provided informed consent and use of samples for research was approved by ethical review boards at the Walter Reed Army Institute of Research, USA, Chulalongkorn University Faculty of Medicine, Thailand and Advarra, USA.

Note that full information on the approval of the study protocol must also be provided in the manuscript.

Field-specific reporting

Please select the one below that is the best fit for your research. If you are not sure, read the appropriate sections before making your selection.

☒ Life sciences ☐ Behavioural & social sciences ☐ Ecological, evolutionary & environmental sciences

For a reference copy of the document with all sections, see nature.com/documents/nr-reporting-summary-flat.pdf

Life sciences study design

All studies must disclose on these points even when the disclosure is negative.

Sample size	Sample size was selected based on availability of PBMC from the two clinical cohorts.
Data exclusions	One outlier was removed as part of the interaction analysis in Figure 2F-G that did not meet assumptions of the linear models and is detailed in the methods section.
Replication	Findings from the discovery study were replicated in an independent cohort. Reproducibility of other experiments was performed by running multiple samples with appropriate controls.
Randomization	All participants contracted HIV-infection in this study and randomization was not performed as it is not a clinical study. Other experimental data included controls and randomization was not necessary.
Blinding	Blinding was not relevant as there was no placebo group.

Reporting for specific materials, systems and methods

We require information from authors about some types of materials, experimental systems and methods used in many studies. Here, indicate whether each material, system or method listed is relevant to your study. If you are not sure if a list item applies to your research, read the appropriate section before selecting a response.

Materials & experimental systems

n/a	Involved in the study
☐	☒ Antibodies
☐	☒ Eukaryotic cell lines
☒	☐ Palaeontology and archaeology
☒	☐ Animals and other organisms
☒	☐ Clinical data
☒	☐ Dual use research of concern
☒	☐ Plants

Methods

n/a	Involved in the study
☒	☐ ChIP-seq
☐	☒ Flow cytometry
☒	☐ MRI-based neuroimaging

Antibodies

Antibodies used

All antibodies are included in supplementary data file 3 and includes the following information: clone, manufacturer, catalog #, the dilutions used, and RRIDs when available.

Validation

The antibodies used are well known clones that are validated by the manufacturer. Each antibody was titrated in house, with normal PBMC, using an 8-point, 2-fold dilution to determine the optimal concentration and signal to noise ratio and to ensure the antibody functioned as expected. Each lot was individually tested.

Eukaryotic cell lines

Policy information about cell lines and Sex and Gender in Research

Cell line source(s)

A549-Dual Cells, human epithelial cells derived from this lung carcinoma cell line, were obtained from InvivoGen (cat# s549d-nfis).
HEK293T human embryonic kidney cells were obtained from ATCC (accession# CRL-3216).
TZM-bl reporter cells were originally derived from the human HeLa cell line, and were obtained from BEI Resources (accession# ARP-8129).
ACH-2 cells, derived from A3.01 cells and latently infected with HIV-1, were obtained from BEI Resources (accession# ARP-349).

Authentication

The cell lines were authenticated by ATCC, or their lab of origin, and not validated further in our laboratory. Specifications of authentification are available from the manufacturer or laboratories of origin.

Mycoplasma contamination

These cell lines were originally obtained as mycoplasma-free. Cells were subsequently tested routinely using a PCR based test and shown to be free of mycoplasma.

Commonly misidentified lines
(See ICLAC register)

None

Plants

Seed stocks

Report on the source of all seed stocks or other plant material used. If applicable, state the seed stock centre and catalogue number. If plant specimens were collected from the field, describe the collection location, date and sampling procedures.

Novel plant genotypes

Describe the methods by which all novel plant genotypes were produced. This includes those generated by transgenic approaches, gene editing, chemical/radiation-based mutagenesis and hybridization. For transgenic lines, describe the transformation method, the number of independent lines analyzed and the generation upon which experiments were performed. For gene-edited lines, describe the editor used, the endogenous sequence targeted for editing, the targeting guide RNA sequence (if applicable) and how the editor was applied.

Authentication

Describe any authentication procedures for each seed stock used or novel genotype generated. Describe any experiments used to assess the effect of a mutation and, where applicable, how potential secondary effects (e.g. second site T-DNA insertions, mosiacism, off-target gene editing) were examined.

Flow Cytometry

Plots

Confirm that:

☒ The axis labels state the marker and fluorochrome used (e.g. CD4-FITC).

☒ The axis scales are clearly visible. Include numbers along axes only for bottom left plot of group (a 'group' is an analysis of identical markers).

☒ All plots are contour plots with outliers or pseudocolor plots.

☒ A numerical value for number of cells or percentage (with statistics) is provided.

Methodology

Sample preparation

Multiparameter flow cytometry: PBMC from 14 participants were stained with Aqua Live/Dead stain (cat# L34957), washed, and blocked using normal mouse IgG (cat# 10400C) (both ThermoFisher). The cells from each participant were then split into four to run four different polychromatic flow panels using conjugated fluorescently labeled monoclonal antibodies against several surface markers to define B, T, Myeloid, and NK cell subsets (Supplementary data file 2-3).

Flow cytometry staining of pMorpheus infection: PBMC from healthy participants were isolated by using separation medium, stimulated for 3 days with PHA (2 μg/ml), and cultured in RPMI 1640 medium with 10% fetal calf serum and 10 ng/ml IL-2 prior to infection. Some of the cells were treated with IL1B 2 days before pMorpheus infection, some at the time of infection, and some 2 days after. Five days postinfection, cells were collected and surface-stained against V5 (V5 Alexa Fluor 647, ThermoFisher; cat# 451098). After 30 min incubation, cells were washed 3x with PBS, fixed with 4% PFA (ChemCruz; cat# sc-281692) for 30 min, and analyzed by flow cytometry (FACSCanto; BD BioSciences).

In vitro functional characterization: Effects of IL1B on cell population frequencies and HIV infection: PBMC were isolated from the blood of healthy participants by density centrifugation on a Ficoll-Paque Plus (GE Healthcare; cat# GE17144002) gradient and stimulated by anti-CD3/CD28 Dynabeads (Gibco; cat# 11132D) at a 1:1 ratio with the estimated CD4+ T cell population in PBMC (25% in total PBMC) in Complete Cell Culture Medium (RPMI 1640 Medium with GlutaMAX and HEPES (cat# 72400047), 10% fetal bovine serum (cat# A3840001), and penicillin/streptomycin (cat# 15140122)) (all Gibco) supplemented with 40 U/ml IL2 (cat# 130-097-743) and with or without recombinant IL1B (cat# 130-095-374) (both Miltenyi Biotec) at four different concentrations (0.01-10 ng/ml, at 10-fold intervals) for 4 days. Treated PBMC were either immediately analyzed using a FACSymphony A5 (BD BioSciences) to assess frequencies of T cell subpopulations, or infected with an R5 tropic molecular clone, YU-2, at a concentration of 1 μg of p24 per million cells and cultured for a further 2 days before assessing the relative frequencies of infected cells by flow cytometry (BD BioSciences).

Instrument

Multiparameter flow cytometry: BD FACS Symphony A5.
Flow cytometry staining of pMorpheus infection: BD FACS Canto.
In vitro functional characterization: BD FACS Symphony A5.

Software

Multiparameter flow cytometry: Data were analyzed with FlowJo v.9.9.6 or higher (Becton Dickinson).
Flow cytometry staining of pMorpheus infection: FACs Diva; analysis FlowJo (Becton Dickinson).
In vitro functional characterization: Data were analyzed with FlowJo v.9.9.6 or higher (Becton Dickinson).

Cell population abundance

Multiparameter flow cytometry: Not Applicable
Flow cytometry staining of pMorpheus infection: Not Applicable
In vitro functional characterization: A total of approximately one million cells per sample were scanned, with a minimum of 500,000 cells per sample across all experiments. The proportion of CD3+ cell populations within live lymphocytes was consistently higher than 95%, with CD4+ T cells ranging between 40% and 60% within the CD3+ population.

Gating strategy

Multiparameter flow cytometry: Included in the supplementary data.
Flow cytometry staining of pMorpheus infection: Live population was gated out in FSC-A and SSC-A; doublets were gated out in the FSC-H and FSC-A. The boundaries between double stained and single stained/expressing cells were set using compensation with single stained beads.
In vitro functional characterization: Lymphocytes were identified using FSC-A and SSC-A. Single cells were isolated based on linear regression of FSC-A and FSC-H parameters. Live cells were gated as the Aqua Live/Dead dye-negative population. From these, CD3+ CD4+ double-positive cells were gated, and CD8+ populations were subsequently excluded.

☒ Tick this box to confirm that a figure exemplifying the gating strategy is provided in the Supplementary Information.
